# Influence of Adjuvant Radiotherapy Timing on Survival Outcomes in High-Risk Patients Receiving Neoadjuvant Treatments

**DOI:** 10.3389/fonc.2022.905223

**Published:** 2022-07-15

**Authors:** Lu Cao, Cheng Xu, Meng-Di Wang, Wei-Xiang Qi, Gang Cai, Rong Cai, Shu-Bei Wang, Dan Ou, Min Li, Kun-Wei Shen, Jia-Yi Chen

**Affiliations:** ^1^ Department of Radiation Oncology, Ruijin Hospital, Shanghai Jiaotong University School of Medicine, Shanghai, China; ^2^ Comprehensive Breast Health Center, Ruijin Hospital, Shanghai Jiaotong University School of Medicine, Shanghai, China

**Keywords:** breast cancer, adjuvant radiotherapy, time to initiation of adjuvant radiotherapy, neoadjuvant treatment, restricted cubic splines

## Abstract

**Purpose:**

To determine the relationship between time to radiotherapy (TTR) and survival outcomes in breast cancer (BC) patients treated with neoadjuvant treatments (NATs).

**Methods:**

Continuous non-metastatic BC patients receiving NAT and adjuvant radiotherapy (RT) from 2009 to 2016 were retrospectively reviewed. A multivariable Cox model with restricted cubic splines (RCSs) was used to determine the panoramic relationship between TTR and survival outcomes. Multivariable analysis was used to control for confounding factors between the groups of TTR.

**Results:**

A total of 315 patients were included. The RCS modeling demonstrated a non-linear relationship between TTR and survival outcomes. The lowest risk for distant metastasis-free survival (DMFS) and recurrence-free survival (RFS) was observed at the TTR of 12 weeks, and the lowest risk of BC-specific survival (BCSS) at 10 weeks. TTR was accordingly transformed into categorical variables as ≤10, 11–20, and >20 weeks. Multivariable analysis revealed that the TTR of ≤10 weeks was an independent prognostic factor for worse DMFS (HR = 2.294, 95% CI 1.079–4.881) and RFS (HR = 2.126, 95% CI 1.038–4.356) compared with the TTR of 10–20 weeks, while the is no difference in DMFS, RFS, and BCSS between TTR >20 weeks and TTR of 10–20 weeks.

**Conclusion:**

There exists a non-linear relationship between TTR after surgery and survival outcomes in patients treated with NAT. Early initiation of RT following surgery does not seem to be associated with a better therapeutic outcome. A relatively flexible recommendation of TTR could be adopted in clinical practice.

## Introduction

Several randomized trials have shown similar survival outcomes with the use of neoadjuvant treatment (NAT) compared with adjuvant chemotherapy; thus, NAT has been increasingly accepted in patients with invasive breast cancer (BC) indicated for adjuvant chemotherapy (1). The majority of patients also have indications for adjuvant radiotherapy (RT) following NAT. However, the optimal time to RT (TTR) after surgery remains controversial in clinical practice and trials (2).

Compared with those who received upfront surgery, patients indicated for NAT often have advanced-stage disease and therefore, a timely initiation of adjuvant RT would be more important, considering the high burden of subclinical diseases. However, it has been found that the immunosuppression status exists after the double whammy of NAT and radical surgery (3, 4). The optimal timing of RT delivery must be integrated with patients’ general condition as well as multidisciplinary treatment to provide the best therapeutic efficacy without further compromising the impaired immune status (5). The recently published KATHERINE study (NCT01772472) and CREATE-X trial (UMIN000000843) have proven the efficacy of additional adjuvant systemic treatment in patients with residual invasive BC after NAT; however, a substantial difference existed in restriction on the timing of RT between these two trials (6, 7). The initiation of RT was required to be within 60 days after surgery in the KATHERINE study, while the competition of RT was allowed as late as 120 days after surgery or the completion of adjuvant capecitabine in the CREATE-X trial. Both restrictions are more empirical than evidence based, which also limits their reference value in guiding clinical practice. Confusion persists for clinicians in determining the optimal TTR for patients following NAT.

For ethical reasons, it is impossible to carry out randomized controlled trials. Hence, we conducted this study to evaluate the panoramic relationship between TTR and survival outcomes in patients treated with NAT using a multivariable Cox model with restricted cubic splines (RCSs), with the purpose to provide evidence for determining optimal TTR in clinical practice.

## Methods and Materials

### Patients

The medical records of consecutive non-metastatic BC patients treated with NAT and definitive surgery, followed by RT, from January 2009 to December 2016 in our institution were retrospectively reviewed. All patients underwent the ultrasonography-guided diagnostic core biopsy of primary tumor before NAT; fine needle aspiration was used to determine the nodal status in case of a positive ultrasound finding. The study was approved by the Medicine Review Board of our institution, and a waiver of consent was obtained.

The status of the estrogen receptor (ER), progesterone receptor (PR), human epidermal growth factor receptor 2 (HER2), and histological grade was obtained from a diagnostic core biopsy. ER and PR statuses were assessed by immunohistochemical analysis (IHC). The percentage of cells staining ER or PR positive >1% was considered hormone receptor (HR) positive. The status of HER2 was defined as positive by an expression level intensity of 3+ on IHC or a gene amplification ratio greater than 2.2 by fluorescence *in situ* hybridization. Molecular subtypes were identified by the ER, PR, and HER2 statuses according to the St Gallen International Expert Consensus (8). The assignment of points for Neo-Bioscore staging was determined for each patient according to the previously published study by Mittendorf et al. to have additional prognostic information (9). Response to NAT was evaluated with reference to the Miller–Payne grading system (10) and revised new response evaluation criteria in solid tumor (RECIST, version 1.1) (11).

### Treatments

The systemic treatment strategies were determined at a multidisciplinary team meeting as we have previously described (12). NAT was administered mainly using a combination of anthracycline and taxane regimes. Adjuvant chemotherapy was usually given to patients with residual invasive tumors in breast or lymph nodes. Adjuvant endocrine therapy was given to patients with HR-positive tumors and often after the completion of RT. Targeted therapy was recommended for patients with HER2-positive disease.

Dose prescription to whole breast/chest wall and regional nodes (supraclavicular, infraclavicular with or without internal mammary nodes) was 50 Gy in 25 fractions. A sequential tumor bed boost of 10–16 Gy in 5–8 fractions was delivered to patients treated with breast-conserving surgery (BCS). The decision of regional nodal irradiation (RNI) was at the discretion of the radiation oncologist, usually based on clinical positive lymph nodes, unfavorable response to NAT, or unfavorable biomarkers. Radiation technique to the breast, tumor bed, chest wall, and regional nodes was consistent as previously reported (13, 14). The volume delineation and definition were determined according to the Radiation Therapy Oncology Group guidelines (15).

### Restricted Cubic Splines and Stratification of the Cohort by Time to Radiotherapy

TTR was defined as the time interval between the date of definitive breast surgery to the initiation of RT. Locoregional recurrence (LRR) was defined as any first recurrence within the ipsilateral chest wall, breast, or regional nodes. All recurrences at distant sites were recorded as distant metastasis (DM). Recurrence-free survival (RFS) was calculated from the initiation date of RT to the first date of LRR, DM, contralateral BC, or BC death, whichever occurred first. BC-specific survival (BCSS) was calculated from the initiation date of RT to deaths from BC.

To model the relationship between TTR and survival outcomes, we developed a multivariable Cox model with RCSs (16–19). The Cox model built was adjusted for comorbidity (including diabetes, hypertension, cardiovascular diseases, endocrine disorder, chronic renal insufficiency, chronic respiratory disease, and autoimmune disease), clinical T (cT) stage, clinical N (cN) stage, histological grade, HR status, HER2 status, Ki67 (as continuous), response to NAT, the type of primary surgery, the administration of adjuvant chemotherapy, targeted therapy, and the delivery of internal mammary node (IMN) RT. The spline was defined using four knots at the 5th, 35th, 65th, and 90th percentiles. The thresholds were determined as timepoints with the smallest and largest hazard ratios (HRs). Because the non-linear P-value for LRR-free survival (LRRFS) was 0.112, no RCS modeling of TTR and LRRFS was further built. Based on the thresholds derived from RCS modeling, TTR was transformed into categorical variables as ≤10, 11–20, and >20 weeks. The interval of 11–20 weeks was considered the reference variable in further analyses using the Cox regression model and propensity score matching (PSM).

### Statistical Analysis

One-to-one PSM was performed to eliminate the selection bias of TTR after surgery (≤ 10, 11–20, and > 20 weeks). Patients were matched on comorbidity, ypT stage, ypN stage, histological grade, HR status, and HER2 status. The caliper width used was equal to 0.2 of the standard deviation of the logit of the propensity score (20). The differences in survival outcomes between TTR groups were further confirmed in the matched cohorts, which were detailed in the **supplemental materials**.

Differences between groups of TTR were assessed by the chi-square test or Fisher exact test for categorical variables and Kruskal–Wallis H test for continuous variables. Survival curves were estimated using the Kaplan–Meier method and compared by the log-rank test. Univariable analyses of potential risk factors for survival outcomes were conducted using a Cox regression model. After adjusting for potential confounding factors (factors related to TTR and prognostic factors identified in univariable analyses and well-established parameters), the independent impact of TTR was tested using a Cox regression model for multivariate analysis. HR and 95% confidence limits (CIs) were presented. All tests were two sided; a P-value of less than 0.05 was considered statistically significant. Statistical analyses were performed using SPSS software version 25.0 (IBM Corporation, Armonk, New York, USA), R version 3.6.3 (R Foundation), and GraphPad Prism 6 (GraphPad Software, La Jolla, CA, USA).

## Results

### Patients and Treatments

**Figure 1:**
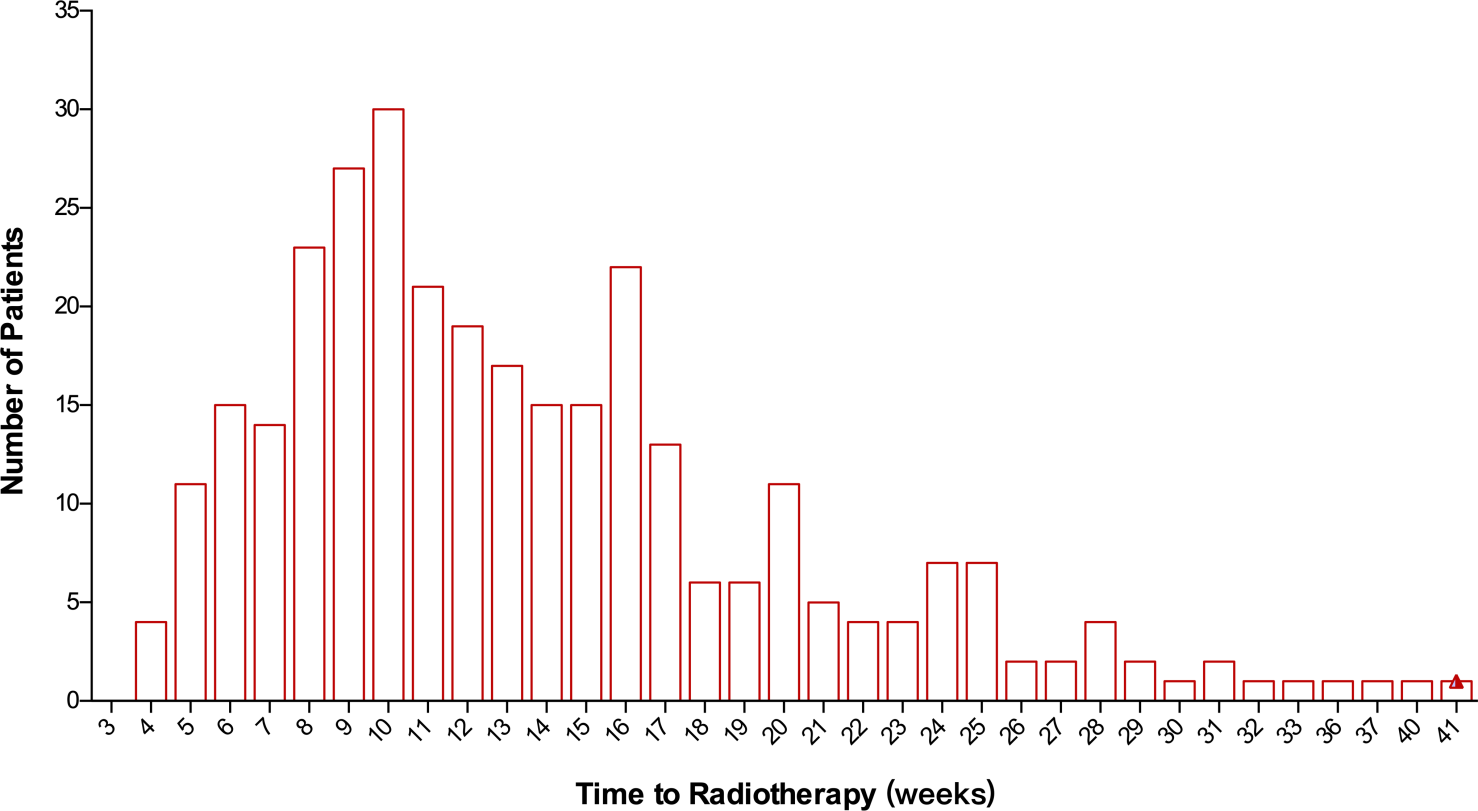
Distribution of patients according to the time to initiation of radiotherapy following surgery.

In total, 315 patients were included in this study. The median TTR was 12 (range: 4–44) weeks. There were 60.1% of patients who started RT between 8 and 16 weeks after surgery (**Figure 1**). Median TTR was 16 (range: 5–41) weeks and 10 (range: 4–31) weeks in patients with or without adjuvant chemotherapy, respectively. Among 178 patients with no adjuvant chemotherapy, there were 32.6% (N=58), 58.3% (N=104), and 9.1% (N=16) of patients who began RT within 8 weeks, 8–16 weeks, and >16 weeks from surgery, respectively. For 120 patients receiving adjuvant chemotherapy before RT, the median interval between chemotherapy and the initiation of RT was 5 (range: 1–23) weeks and 92.7% of patients started RT within 12 weeks after the last chemotherapy. Among 21 patients with non-pCR and receiving adjuvant capecitabine, the median TTR was 13 (range: 5–40) weeks. The main reasons for RT delay include shoulder dysfunction, delayed wound healing, chemotherapy-related toxicity, delayed clinic visits, and a waiting list for RT.

In total, 289 out of 315 patients completed a full course of NAC as planned. All patients were with negative margin. Among 115 patients with HER2-positive tumors, 89 (77.4%) received the (neo)adjuvant trastuzumab. All patients with HR-positive tumors were treated with endocrine therapy. The proportion of comorbidity, ypT stage, ypN stage, histological grade, NAT regimens, the receipt of adjuvant chemotherapy, and adjuvant chemotherapy regimens vary significantly among the three groups of TTR (all P-values <0.05). Significantly fewer patients with TTR ≤10 weeks were treated with adjuvant chemotherapy compared with patients with a TTR of 10–20 weeks and those with a TTR >20 weeks (19.4% vs. 53.1% vs. 78.3%, P-value < 0.01). The patients and treatment characteristics of the whole cohort are detailed in **Table 1**. In the matched cohorts, the prognostic factors of the clinical stage, pathological stage, and tumor markers of the HR status, HER2 status, and histological grade were balanced between the group of TTR ≤10 weeks (N=95) and group of TTR of 10–20 weeks (N=95) and between the group of TTR >20 weeks (N=36) and the group of TTR of 10–20 weeks (N=36) (**Table S1**).

**Table 1:** Patients and treatment characteristics by time to radiotherapy (TTR) among patients treated with neoadjuvant treatments (NATs).

Characteristics	All patients (N=315)	TTR (weeks)			P-value
		≤10 (N=124)	11–20 (N=145)	>20 (N=46)	
	N (%)	N (%)	N (%)	N (%)	
**Age** (years)					
Median (range)	51 (23-87)	49 (23-79)	53 (25-87)	49.5(28-75)	0.08
**Menopausal status**					
Pre/perimenopausal	157 (49.8)	68 (54.8)	65 (44.8)	24 (52.2)	0.25
Menopausal	158 (50.2)	56 (45.2)	80 (55.2)	22 (47.8)	
**Comorbidity**					
No	244 (77.5)	100 (80.6)	103 (71)	41 (89.1)	0.02
Yes	71 (22.5)	24 (19.4)	42 (29)	5 (10.9)	
**cT stage**					
T1	58 (18.4)	19 (15.3)	32 (22.1)	7 (15.2)	0.60
T2	186 (59)	72 (58.1)	83 (57.2)	31 (67.4)	
T3	46 (14.6)	21 (16.9)	19 (13.1)	6 (13)	
T4	25 (7.9)	12 (9.7)	11 (7.6)	2 (4.3)	
**cN stage**					
N0	39 (12.4)	12 (9.7)	21 (14.5)	6 (13)	0.49
N1	152 (48.3)	57 (46)	69 (47.6)	26 (56.5)	
N2	93 (29.5)	40 (32.3)	44 (30.3)	9 (19.6)	
N3	31 (9.8)	15 (12.1)	11 (7.6)	5 (10.9)	
**ypT stage**					
T0-is	63 (20)	30 (24.2)	29 (20)	4 (8.7)	<0.01
T1	128 (40.6)	52 (41.9)	64 (44.1)	12 (26.1)	
T2	97 (30.8)	29 (23.4)	41 (28.3)	27 (58.7)	
T3	21 (6.7)	10 (8.1)	8 (5.5)	3 (6.5)	
T4	6 (1.9)	3 (2.4)	3 (2.1)	0 (0)	
**ypN stage**					
N0	109 (34.6)	48 (38.7)	54 (37.2)	7 (15.2)	0.045
N1	85 (27)	29 (23.4)	42 (29)	14 (30.4)	
N2	69 (21.9)	24 (19.4)	30 (20.7)	15 (32.6)	
N3	52 (16.5)	23 (18.5)	19 (13.1)	10 (21.7)	
**Histological grade**					
I	9 (2.9)	5 (4)	3 (2.1)	1 (2.2)	0.01
II	149 (47.3)	44 (35.5)	83 (57.2)	22 (47.8)	
III	157 (49.8)	75 (60.5)	59 (40.7)	23 (50)	
**HR status**					
Negative	132 (41.9)	61 (49.2)	54 (37.2)	17 (37)	0.08
Positive	183 (58.1)	63 (50.8)	91 (62.8)	29 (63)	
**HER2 status**					
Negative	200 (63.5)	79 (63.7)	91 (62.8)	30 (65.2)	0.95
Positive	115 (36.5)	45 (36.3)	54 (37.2)	16 (34.8)	
**Ki67 (%)**					
Median (IQR)	30(15-60)	50 (15-70)	30 (15-60)	30 (15-60)	0.43
≤14	63 (20)	23 (18.5)	32 (22.1)	30 (65.2)	0.84
>14	221 (70.2)	88 (71)	103 (71)	16 (34.8)	
Missing data	31 (9.8)	13 (10.5)	10 (6.9)	8 (17.4)	
**Molecular subtype**					
Luminal	131 (41.6)	48 (38.7)	62 (42.8)	21 (45.7)	0.838
TNBC	69 (21.9)	31 (25)	29 (20)	9 (19.6)	
HER2 positive	115 (36.5)	45 (36.3)	54 (37.2)	16 (34.8)	
**Neo-Bioscore score**					
Median (range)	3 (0-7)	4 (0-7)	3 (1-6)	3.5 (1-6)	
1–3	168 (53.3)	59 (47.6)	86 (59.3)	23 (50)	0.14
4–6	147 (46.7)	65 (52.4)	59 (40.7)	23 (50)	
**Type of primary surgery**					
Mastectomy	271 (86)	101 (81.5)	127 (87.6)	43 (93.5)	0.10
BCS	44 (14)	23 (18.5)	18 (12.4)	3 (6.5)	
**NAT regimens**					
Taxanes	44 (14)	15 (12.1)	24 (16.6)	5 (10.9)	<0.01
Anthracycline	46 (14.6)	1 (0.8)	26 (17.9)	19 (41.3)	
Taxanes + anthracycline	214 (67.9)	107 (86.3)	87 (60)	20 (43.5)	
Endocrine therapy	11 (3.5)	1 (0.8)	8 (5.5)	2 (4.3)	
**Response to NAT**					
pCR	48 (15.2)	25 (20.2)	21 (14.5)	2 (4.3)	0.04
PR	233 (74)	85 (68.5)	111 (76.6)	37 (80.4)	
SD	21 (6.7)	8 (6.5)	9 (6.2)	4 (8.7)	
PD	13 (4.1)	6 (4.8)	4 (2.8)	3 (6.5)	
**Adjuvant chemotherapy**					
No	178 (56.5)	100 (80.6)	68 (46.9)	10 (21.7)	<0.01
Yes	137 (43.5)	24 (19.4)	77 (53.1)	36 (78.3)	
**Adjuvant chemotherapy regimens**					
Taxanes	59 (18.7)	2 (1.6)	41 (28.3)	16 (34.8)	<0.01
Anthracycline	11 (3.5)	0 (0)	9 (6.2)	2 (4.3)	
Taxanes + anthracycline	39 (12.4)	10 (8.1)	15 (10.3)	14 (30.4)	
Other	28 (8.9)	12 (9.7)	12 (8.3)	4 (8.7)	
**Targeted therapy in HER2+**					
No	26 (22.6)	9 (20)	12 (22.2)	5 (31.3)	0.65
Yes	89 (77.4)	36 (80)	42 (77.8)	11 (68.8)	
**RNI**					
No	18 (5.7)	5 (4)	12 (8.3)	1 (2.2)	0.18
Yes	297 (94.3)	119 (96)	133 (91.7)	45 (97.8	
**IMN RT**					
No	160 (50.8)	62 (50)	77 (53.1)	21 (45.7)	0.66
Yes	155 (49.2)	62 (50)	68 (46.9)	25 (54.3)	

TTR, time to radiotherapy; HR, hormone receptor; HER2, human epidermal growth factor receptor 2, IQR, interquartile range, BCS, breast-conserving surgery; NAT, neoadjuvant treatment; pCR, pathological complete response; PR, partial response; SD, stable disease; PD, progressive disease; RNI, regional nodal irradiation; IMNs, internal mammary nodes; RT, radiotherapy.

### RCS Modeling

**Figure 2:**
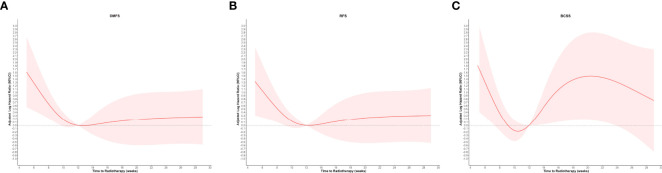
Restricted cubic spline modeling of the relationship between the time to initiation of adjuvant radiotherapy and survival outcomes of distant metastasis-free survival (DMFS), recurrence-free survival (RFS), and breast cancer–specific survival (BCSS). The log of the hazard ratios (HRs) derived from a multivariate Cox regression model is shown on the y-axis. The 95% Cis of the adjusted HRs are represented by the shaded area. **(A)** DMFS; **(B)** RFS; and **(C)** BCSS.

The graphical visualization of RCS modeling for DMFS, RFS, and BCSS is shown in **Figure 2**. The risk function of RCS modeling demonstrated a non-linear relationship between TTR and survival outcomes. At the starting point of TTR (4 weeks after surgery), the risk of DM, any recurrence, and BCSS were all at a high level and then continuously declined with increasing TTR. Beyond the time point of 10 weeks, the risk of BCSS switched to increase and reached the top at the time point of 20 weeks and then decreased again slowly with the extension of TTR. For DMFS and RFS, the log HR curves remained flat with the extension of TTR after the time point of 12 weeks.

### Pattern of Recurrences

The pattern of failure stratified by the groups of TTR is detailed in **Table 2**. During follow-up, there were 18 and 66 patients who developed LRR and DM as first recurrence events, respectively. There was a significant difference in the rate of DM among the three TTR groups, with 28.2% in TTR ≤10 weeks, 15.9% in the TTR of 10 –20 weeks, and 32.6% in TTR >20 weeks (P-value = 0.013). In the matched cohort, the rate of DM was also significantly higher in TTR ≤10 weeks compared with the TTR of 10–20 weeks (14.7% vs. 26.3%, P-value = 0.044).

**Table 2:** Pattern of failures by TTR among patients treated with NATs.

	All patients	TTR (weeks)			P-value
		≤ 10	11-20	>20	
	N (%)	N (%)	N (%)	N (%)	
**Whole cohort**	**N = 315**	**N = 124**	**N = 145**	**N = 46**	
**Locoregional recurrence**	18 (5.7)	11(8.9)	5 (3.4)	2 (4.3)	0.137
Chest wall	7 (2.2)	3 (2.4)	3 (2.1)	1 (2.2)	
Supraclavicular LN	4 (1.3)	2 (1.6)	2 (1.4)	0 (0)	
Axillary LN	2 (0.6)	1 (0.8)	0 (0)	1 (2.2)	
Internal mammary LN	1 (0.3)	1 (0.8)	0 (0)	0 (0)	
Multisite	4 (0.13)	4 (3.2)	0 (0)	0 (0)	
**Distant metastasis**	73 (23.2)	35 (28.2)	23 (15.9)	15 (32.6)	0.013
As first events	66 (20.9)	32 (25.8)	20 (13.8)	14 (30.4)	0.013
**Matched cohort 1**	**N = 190**	**N = 95**	**N = 95**		
**Locoregional recurrence**	10 (5.3)	7 (7.3)	3 (3.1)		0.213
**Distant metastasis**	39 (20.5)	25 (26.3)	14 (14.7)		0.044
DM as first event	35 (18.4)	23 (12.6)	12 (12.6)		0.04
**Matched cohort 1**	**N = 72**		**N = 36**	**N = 36**	
**Locoregional recurrence**	3 (4.2)		2 (5.5)	1 (2.7)	0.555
**Distant metastasis**	20 (27.8)		7 (19.4)	13 (36.1)	0.131
DM as first event	20 (27.8)		7 (19.4)	13 (36.1)	0.131

LN, lymph nodes; DM, distant metastasis.

### Survival Outcomes

**Figure 3:**
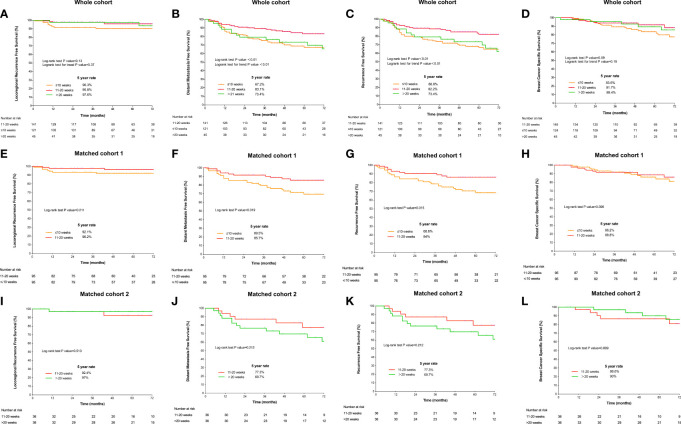
Survival curves according to the time to initiation of adjuvant radiotherapy estimated by the Kaplan–Meier method and compared using the log-rank test in the whole cohort. **(A)** locoregional RFS; **(B)** distant metastasis-free survival; **(C)** recurrence-free survival; and **(D)** overall survival and in matched cohorts. Comparison of survival outcomes between the TTR groups of 11–20 weeks and >20 weeks: **(E)** locoregional RFS; **(F)** DMFS; **(G)** RFS; and **(H)** BCSS. Comparison of survival outcomes between the TTR groups of 11–20 weeks and ≤10 weeks: **(I)** locoregional recurrence-free survival; **(J)** DMFS; **(K)** RFS; and **(L)** BCSS.

The median follow-up was 54 (interquartile range: 38–75) months. Five-year LRRFS, DMFS, RFS, and BCSS were 93.9%, 75.4%, 75.3%, and 88.1%, respectively. Compared with TTR ≤10 weeks or TTR >20 weeks, the TTR of 10–20 weeks had better 5-year DMFS (67.2% or 73.4%, vs. 83.1%, P-value <0.01; **Figure 3B**) and 5-year RFS (66.6% or 73.4%, vs. 82.2%, P-value <0.01; **Figure 3C**). In the matched cohort, the TTR of 10–20 weeks was significantly associated with an improved 5-year rate of DMFS (85.7% vs 69.5%, P-value = 0.019) and RFS (84% vs 68.6%, P-value = 0.015) compared with TTR ≤10 weeks (**Figures 3F, G**). The comparisons of survival outcomes between TTR groups in the whole cohort and the matched cohorts are shown in **Figure 3**.

On univariable Cox regression analysis, the TTR of 10–20 weeks was consistently associated with significantly favorable DMFS and RFS (P-values <0.05). A significantly worse BCSS was found in TTR ≤10 weeks compared with the TTR of 10–20 weeks (HR = 2.168, 95% CI 1.073–4.383, P-value = 0.03). The univariable analyses of potential risk factors for survival outcomes are detailed in **Table S2**.

In multivariable analysis, TTR ≤10 weeks remained as an independent unfavorable prognostic factor for DMFS (HR = 2.294, 95% CI 1.079–4.881, P-value = 0.03) and RFS (HR = 2.126, 95% CI 1.038–4.356, P-value = 0.04) compared with the TTR of 10–20 weeks. However, no significant difference in the risk of survival outcomes was detected between the TTR of 10–20 weeks and TTR >20 weeks after adjusting for potential confounding factors. In addition, the factors of cT, cN, ypN, HR status, and the administration of adjuvant chemotherapy were also found to independently predict survival outcomes (P-values < 0.05). The multivariable models in the whole cohort and the matched cohorts are detailed in **Table 3** and **Table S3**, respectively.

**Table 3:** Adjusted hazard ratio (HR) of survival outcomes by TTR in the multivariable Cox proportional hazards model.

	LRRFS			DMFS			RFS			BCSS		
Parameters	HR	95% CI	P- value	HR	95% CI	P- value	HR	95% CI	P value	HR	95% CI	P- value
**TTR (weeks)**												
11-20	1	–		1	–		1	–		1	–	
≤ 10	2.24	0.57-8.79	0.248	2.19	1.15-4.14	0.016	2.13	1.14-3.96	0.017	1.98	0.8-4.85	0.138
>20	0.58	0.09-3.66	0.561	1.41	0.68-2.89	0.351	1.29	0.62-2.64	0.491	1	0.33-2.96	0.999
**cT stage**												
T1							1	–				
T2							2.36	1.02-5.44	0.044			
T3							2.16	0.77-6.03	0.142			
T4							3.08	0.96-9.79	0.057			
**cN stage**												
N0				1	–							
N1				1.19	0.43-3.3	0.736						
N2				1.66	0.57-4.79	0.352						
N3				3.27	1.05-10.09	0.04						
**ypN stage**												
N0				1	–		1	–				
N1				0.96	0.36-2.5	0.925	0.99	0.39-2.51	0.984			
N2				2.26	0.9-5.65	0.083	2.55	1.03-6.25	0.041			
N3				3.51	1.41-8.71	0.007	3.67	1.5-8.95	0.004			
**HR status**												
Negative	1	–	0.006				1	–	0.018	1	–	<0.001
Positive	0.09	0.01-0.5					0.47	0.25-0.88		0.16	0.06-0.39	
**Adjuvant chemotherapy**												
No	1	–	0.025									
Yes	4.96	1.22-20.15										

Variables in the model include cT, cN, ypT, ypN, histological grade, HR, HER2, response to NAC, the type of primary surgery, the administration of adjuvant chemotherapy, targeted therapy, and the delivery of IMN RT.

Impact on survival outcomes among subgroups stratified by clinical stage, ypN stage, molecular subtypes, and the type of primary surgery (**Table 4**) was further analyzed. After adjusting for all confounders, in patients with clinical stage III, TTR ≤ 10 weeks was associated with significantly worse DMFS (HR = 3.177, 95% CI 1.247–8.094, P-value = 0.015) and RFS (HR = 3.416, 95% CI 1.316–8.868, P-value = 0.012) compared with the TTR of 10–20 weeks. Similar results were found in the subgroup of mastectomy. The risk of BCSS was significantly worse in TTR ≤10 weeks compared with the TTR of 10–20 weeks (HR = 234.379, 95% CI 6.863–8004.374, P-value=0.002) among patients with triple-negative BC (TNBC).

**Table 4:** Adjusted HR of survival outcomes by TTR in the multivariable Cox proportional hazards model, stratified by the clinical stage, ypN stage, molecular subtypes, and the type of primary surgery.

TTR (weeks)	No. of patients	LRRFS				DMFS				RFS				BCSS			
		No. of events	HR	95% CI	P- value	No. of events	HR	95% CI	P- value	No. of events	HR	95% CI	P- value	No. of events	HR	95% CI	P- value
**Clinical stage I–II**																	
11–20	75	2	1			9	1			10	1			5	1		
≤ 10	55	1	1.411	/	0.986	9	1.544	0.41-5.73	0.516	10	1.691	0.51-5.56	0.387	8	6.055	0.67-54.54	0.108
>20	29	1	1.731	/	0.978	7	1.157	0.33-4.02	0.819	7	1.136	0.34-3.77	0.836	5	1.537	0.22-10.51	0.661
**Clinical stage III**																	
11–20	70	3	1			14	1			14	1			7	1		
≤ 10	69	10	3.76	0.47-29.67	0.209	26	3.177	1.24-8.09	0.015	27	3.416	1.31-8.86	0.012	14	1.954	0.57-6.59	0.281
>20	17	1	0.416	0.02-6.13	0.523	8	1.443	0.51-4.01	0.482	8	1.472	0.53-4.07	0.456	2	0.282	0.03-2.25	0.233
**ypN0 stage**																	
11–20	55	1	1			3	1			4	1			3	1		
≤ 10	48	1	1	0.03-30.28	1	4	4.303	0.17-105.9	0.372	5	4.509	0.49-40.9	0.181	3	3.833	0.11-131.03	0.456
>20	7	0	1	/	1	1	5.935	0.11-317.41	0.38	1	5.971	0.36-97.6	0.21	0	0.002	/	0.803
**ypN1–3 Stage**																	
11-20	90	4	1			20	1			20	1			9	1		
≤ 10	76	10	2.313	0.45-11.86	0.315	31	1.992	0.98-4.04	0.057	32	1.932	0.95-3.92	0.068	19	1.561	0.52-4.65	0.424
>20	39	2	0.702	0.1-4.92	0.722	14	1.381	0.64-2.94	0.404	14	1.291	0.6-2.77	0.513	7	1.209	0.37-3.91	0.752
**Luminal**																	
11-20	62	2	1			12	1			12	1			5	1		
≤ 10	48	2	0.448	/	0.959	11	1.644	0.56-4.77	0.362	12	2.017	0.7-5.76	0.19	3	1.56	0.21-11.56	0.664
>20	21	1	0.003	/	0.557	10	1.819	0.61-5.42	0.283	10	1.921	0.64-5.73	0.242	4	1.866	0.09-35.06	0.677
**TNBC**																	
11-20	29	2	1			4	1			5	1			4	1		
≤ 10	31	7	2.124	0.15-29.04	0.572	14	4.482	0.45-44.11	0.199	15	1	0.31-3.19	1	12	234.379	6.86-8004.37	0.002
>20	9	0	0.654	0-49.22	0.847	3	1.865	0.1-34.49	0.675	3	1	0.22-4.41	1	1	21.49	0.36-1259.25	0.14
**HER2 positive**																	
11-20	54	1	1			7	1			7	1			3	1		
≤ 10	45	2	1.918	/	0.978	10	7.167	0.26-192.27	0.241	10	0.776	0.02-22.49	0.883	7	243.671	/	0.828
>20	16	1	0.763	/	0.993	2	0.102	0-2.03	0.135	2	0.034	0-1.45	0.185	2	0	/	0.703
**Mastectomy**																	
11-20	127	5	1			23	1			23	1			12	1		
≤ 10	101	10	1.892	0.43-8.31	0.399	33	2.176	1.11-4.23	0.022	35	2.305	1.19-4.46	0.013	22	2.066	0.82-5.17	0.122
>20	43	2	0.469	0.07-2.95	0.42	15	1.388	0.67-2.86	0.375	15	1.315	0.63-2.72	0.461	7	1.002	0.33-2.97	0.997
**BCS**																	
11–20	18	0	1			0	1			1	1			0	/	/	/
≤ 10	23	1	1	/	1	2	1.982	/	0.991	2	0.057	/	0.922	0	/	/	/
>20	3	0	1	/	1	0	0.025	/	0.969	0	0	/	0.846	0	/	/	/

Variables in the model include cT, cN, ypT, ypN, histological grade, HR, HER2, response to NAC, the type of primary surgery, the administration of adjuvant chemotherapy, targeted therapy, and the delivery of IMN RT.

## Discussion

In this study, we explored a dynamic relationship between TTR after surgery and survival outcomes in patients who received NAT. For the first time, a multivariable Cox model using RCS was applied and a non-linear relationship between TTR and survival outcomes of DMFS, RFS, and BCSS was revealed. The lowest risk for DMFS and RFS was observed at 12 weeks after surgery and the lowest risk of BCSS at TTR of 10 weeks. The multivariable analysis confirmed that TTR ≤10 weeks independently predicted significantly compromised DMFS and RFS compared with the TTR of 10–20 weeks, while no difference in survival outcomes between the TTR of 10–20 weeks and TTR >20 weeks were observed.

In our study population, the majority of patients started RT within 12 weeks after the last cycle of adjuvant chemotherapy or within 16 weeks after surgery when no adjuvant chemotherapy is given, which is consistent with previous reports (21–23). In a recent retrospective study of the impact of TTR on survival, among 95 patients treated with NAC without adjuvant chemotherapy, Xie et al. (21) reported that 25 (26.3%), 54 (56.8%), and 16 (16.8%) started RT at <8 weeks, 8–16 weeks, and > 16 weeks from surgery, respectively. Compared with early BC patients treated with breast conservation with no indication of adjuvant chemotherapy, TTR is, in general, longer and more heterogenous in the NAT population. In a large sample study of the impact of RT delay in early-stage patients with no adjuvant chemotherapy, only 24.9% of 186,650 patients started RT >8 weeks after surgery (22).

Before our study, a series of studies have tried to clarify the impact of TTR on survival outcomes and came to different conclusions. In a retrospective study of 6,428 patients treated with BCS and RT without adjuvant chemotherapy, Olivotto et al. (24) found that TTR >20 weeks was associated with inferior LRRFS, DMFS, and BCSS compared with TTR <20 weeks. Nevertheless, no impact of TTR on LRR was observed in a retrospective study of 248 patients receiving NAT and mastectomy using the cut-off points of 8, 12, and 16 weeks (25). Another retrospective study found that early initiating RT after surgery (<42 days) was, on the contrary, associated with worse DMFS in patients treated with BCS and adjuvant chemotherapy (26). In a recent study with the largest sample up to date, Zheleva et al. (27) pointed out that the influence of delayed RT depends on breast primary surgery. In the BCS cohort, delaying RT beyond 365 days after surgery was associated with significantly decreased OS versus timely RT (HR 1.37, 95% CI 1.19–1.58), while such an unfavorable effect was not observed in patients receiving mastectomy. In these previous studies, TTR was either treated as a category variable using predefined cutoff values based on clinical experience or as a continuous variable with the assumption that the relationship between TTR and survival outcomes was linear. These data-processing methods might cause information loss or misinterpretation. To minimize the confounding impact of the data-processing methods of TTR, we applied the multivariable Cox model using RCS, which could explore the panoramic relationship between consecutive changes in TTR and survival outcomes without losing information. Our results showed that the relationship between TTR and survival outcomes was non-linear and dynamically changing. The best therapeutic outcome was observed in patients who started RT 10–12 weeks after surgery. The initiation of RT within 10 weeks after surgery or delaying RT after 20 weeks adversely affected the survival outcomes. This non-linear pattern of relationship between TTR and survival outcomes found in our study might partly explain the inconsistent findings of the impact of TTR after surgery on survival outcomes in previous studies.

Our study is not the first one that reports the negative relationship between short TTR and survival outcomes. Xie et al. (21) found that TTRs of <8 weeks or >16 weeks were associated with increased risks of BC specific mortality and all-cause mortality compared with the TTR of 8–16 weeks. In another study of patients treated with BCS, TTR >55 days was associated with a higher 10-year DFS (HR = 0.60, 95% CI: 0.38–0.94) and DMFS (HR = 0.64, 95% CI: 0.45–0.92) than TTR < 42 days (26). Caponio et al. (28) reported decreased DFS and DMFS when RT was started early using the tertiles of ≤60, 61–120, and >120 days. In a prospective study of 1,070 patients treated with BCS and without adjuvant systemic therapy, better DMFS (HR = 0.3, 95% CI: 0.1–0.8; P-value = 0.017) and BCSS (HR = 0.2, 95% CI: 0.04–0.7; P-value = 0.012) were found for the TTR of 57–112 compared with TTR <45 days (29). In clinical practice, patients with unfavorable prognostic factors may be more likely to be recommended to start RT earlier. To minimize the influence of possible confounding factors, multivariable analysis was conducted with adjusting factors related to TTR and prognostic factors identified in our univariable analyses or well established in previous studies. It was confirmed that TTR ≤10 weeks remains an independent predictor for worse DMFS and RFS. The independent prognostic value of TTR ≤10 weeks was further identified in the matched cohort using multivariable analysis. TTR after surgery is influenced by various factors including response to NAT, the indication of extended adjuvant therapy based on molecular subtypes, and the collaboration of multidisciplinary treatment groups including the waiting list for RT (30–32). To date, the optimal time of RT after surgery has not been clarified. Nevertheless, our results in combination with previous reports cautioned that RT timing after surgery needs careful recommendation instead of a simplified early start. A reasonable and relatively flexible strategy of TTR after surgery could be recommended in clinical practice as well as a reference for prospective study design.

Several reasons might explain the negative impact on survival associated with early initiation of RT. Ionizing irradiation (IR) is a double-bladed sword that could also stimulate invasion and metastasis through the upregulation of key molecules, inducing vascular damage or remodulating the tumor microenvironment (33–35). There is also concern that large fields and fractionated RT could impair host immunologic effects (36). Patients who initiated RT shortly after surgery are more likely to suffer from poor wound healing and incomplete recovery from bone marrow suppression induced by NAT. Our subgroup analyses found that the unfavorable impact of TTR ≤10 weeks was more prominent in patients with clinical stage III and TNBC or receiving mastectomy, which are heavily treated populations. Early initiation of RT in our study is associated with a lower rate of 5-year DMFS and RFS rather than LRRFS echoes our hypothesis. Further preclinical and prospective clinical data are essential to explore the interplay of these factors.

As with all retrospective studies, it is impossible to exclude the heterogeneity of patients’ inclusion. To minimize the confounding impact of selection bias, multivariable analysis using the Cox regression model and PSM analysis was conducted and confirmed the adverse impact of short TTR (<10 weeks) on survival outcomes identified from the RCS modeling. The median follow-up in our study is not sufficient for operable BC even though the general risk of the study population is high. A longer follow-up with the immune microenvironment analysis and a validation study with a prospective database are needed.

## Conclusion

There exists a non-linear relationship between TTR after surgery and survival outcomes in patients treated with NAT with or without adjuvant chemotherapy. Early initiation of RT following surgery does not seem to be associated with a better therapeutic outcome. A relatively flexible recommendation of TTR could be adopted in clinical practice.

## Data Availability Statement

The raw data supporting the conclusions of this article will be made available by the authors, without undue reservation.

## Ethics Statement

All procedures performed in studies involving human participants were in accordance with the ethical standards of the institutional and/or national research committee and with the 1964 Helsinki Declaration and its later amendments or comparable ethical standards.

## Author Contributions

LC and J-YC contributed to the conception, design, and interpretation of the data analysis and were responsible for the drafting of the manuscript. GC, M-DW, RC, S-BW, and DO collected the data. CX and W-XQ analyzed and interpreted the data. ML and K-WS reviewed and edited the manuscript. All authors read and approved the manuscript.

## Funding

This study was supported in part by the National Key Research and Development Program of China (2016YFC0105409), Scientific and Technological Innovation Action Plan of Shanghai Science and Technology Committee (19411950900, 19411950901), Clinical Research Plan of SHDC (SHDC2020CR2052B), and National Natural Science Foundation of China (81602791, 81673102, 81803164, 81972963). The funding sources had no role in study design, data collection, data analysis, data interpretation, or writing of the report.

## Conflict of Interest

The authors declare that the research was conducted in the absence of any commercial or financial relationships that could be construed as a potential conflict of interest.

## Publisher’s Note

All claims expressed in this article are solely those of the authors and do not necessarily represent those of their affiliated organizations, or those of the publisher, the editors and the reviewers. Any product that may be evaluated in this article, or claim that may be made by its manufacturer, is not guaranteed or endorsed by the publisher.
