# Supplementary material for: Influence of Adjuvant Radiotherapy Timing on Survival Outcomes in High-Risk Patients Receiving Neoadjuvant Treatments
Source: Front Oncol. 2022 Jul 15;12:905223. doi: 10.3389/fonc.2022.905223 (PMC9334789; doi:10.3389/fonc.2022.905223)
Supplement: Supplementary file 1 [file Table_1.docx]

**Table S1 Patients and treatment characteristics** **by TTR in the matched cohorts**

|  | **Matched cohort 1** | | |  | **Matched cohort 2** | | |
| --- | --- | --- | --- | --- | --- | --- | --- |
|  | **TTR = 11-20 weeks (N=95)** | **TTR ≤ 10 weeks**  **(N=95)** | **P value** |  | **TTR = 11-20 weeks (N=36)** | **TTR ＞20 weeks (N=36)** | **P value** |
| **Characteristics** | **N (%)** | **N (%)** |  |  | **N (%)** | **N (%)** |  |
| **Age** (years) |  |  |  |  |  |  |  |
| Median (range) | 51 (32-77) | 51 (28-71) |  |  | 51 (25-78) | 49 (23-79) |  |
| **Menopausal status** |  |  |  |  |  |  |  |
| Pre/peri-menopausal | 45 (47.3) | 54 (57.4) | 0.191 |  | 18 (50) | 18 (50) | 0.593 |
| Menopausal | 50 (52.6) | 41 (43.6) |  |  | 18 (50) | 18 (50) |  |
| **Comorbidity** |  |  |  |  |  |  |  |
| No | 76 (80) | 74 (77.8) | 0.859 |  | 33 (91.6) | 31 (86.1) | 0.71 |
| Yes | 19 (20) | 21 (22.1) |  |  | 3 (8.3) | 5 (13.8) |  |
| **cT Stage** |  |  |  |  |  |  |  |
| T1 | 21 (22.1) | 14 (14.7) | 0.635 |  | 9 (25) | 5 (13.8) | 0.506 |
| T2 | 54 (56.8) | 58 (61) |  |  | 18 (50) | 24 (66.6) |  |
| T3 | 13 (13.6) | 12 (12.6) |  |  | 7 (19.4) | 5 (13.8) |  |
| T4 | 7 (7.3) | 11 (11.5) |  |  | 2 (5.5) | 2 (5.5) |  |
| **cN Stage** |  |  |  |  |  |  |  |
| N0 | 13 (13.6) | 8 (8.4) | 0.689 |  | 4 (11.1) | 6 (16.6) | 0.795 |
| N1 | 48 (50.5) | 50 (52.6) |  |  | 19 (52.7) | 20 (55.5) |  |
| N2 | 26 (27.3) | 27 (28.4) |  |  | 9 (25) | 6 (16.6) |  |
| N3 | 8 (8.4) | 10 (10.5) |  |  | 4 (11.1) | 4 (11.1) |  |
| **ypT Stage** |  |  |  |  |  |  |  |
| T0-is | 21 (22.1) | 19 (20) | 0.995 |  | 3 (8.3) | 4 (11.1) | 0.826 |
| T1 | 43 (45.2) | 43 (45.2) |  |  | 11 (30.5) | 11 (30.5) |  |
| T2 | 24 (25.2) | 25 (26.3) |  |  | 19 (52.7) | 20 (55.5) |  |
| T3 | 5 (5.2) | 6 (6.3) |  |  | 2 (5.5) | 1 (2.7) |  |
| T4 | 2 (2.1) | 2 (2.1) |  |  | 1 (2.7) | 0 (0) |  |
| **ypN Stage** |  |  |  |  |  |  |  |
| N0 | 36 (37.8) | 35 (36.8) | 0.874 |  | 6 (16.6) | 7 (19.4) | 0.683 |
| N1 | 28 (29.4) | 24 (25.2) |  |  | 13 (36.1) | 9 (25) |  |
| N2 | 17 (17.8) | 20 (21) |  |  | 12 (33.3) | 12 (33.3) |  |
| N3 | 14 (14.7) | 16 (16.8) |  |  | 5 (13.8) | 8 (22.2) |  |
| **Histological grade** |  |  |  |  |  |  |  |
| I | 2 (2.1) | 5 (5.2) | 0.511 |  | 0 (0) | 1 (2.7) | 0.343 |
| II | 45 (47.3) | 43 (45.2) |  |  | 22 (61.1) | 17 (47.2) |  |
| III | 48 (50.5) | 47 (49.4) |  |  | 14 (38.8) | 18 (50) |  |
| **HR status** |  |  |  |  |  |  |  |
| Negative | 36 (37.8) | 42 (44.2) | 0.461 |  | 9 (25) | 13 (36.1) | 0.443 |
| Positive | 59 (62.1) | 53 (55.7) |  |  | 27 (75) | 23 (63.8) |  |
| **HER2 status** |  |  |  |  |  |  |  |
| Negative | 67 (70.5) | 60 (63.1) | 0.355 |  | 30 (83.3) | 22 (61.1) | 0.064 |
| Positive | 28 (29.4) | 35 (36.8) |  |  | 6 (16.6) | 14 (38.8) |  |
| **Ki67 (%)** |  |  |  |  |  |  |  |
| Median (IQR) | 20 (10-40) | 25 (14-70) |  |  | 30 (20-60) | 40 (15-60) |  |
| ≤ 14 | 16 (18.1) | 19 (22.6) | 0.57 |  | 11 (32.3) | 8 (26.6) | 0.785 |
| >14 | 72 (81.8) | 65 (77.3) |  |  | 23 (67.6) | 22 (73.3) |  |
| missing data | 7 (7.4) | 11 (11.6) |  |  |  |  |  |
| **Molecular subtype** |  |  |  |  |  |  |  |
| Luminal | 49 (51.5) | 40 (42.1) | 0.408 |  | 25 (69.4) | 17 (47.2) | 0.094 |
| TNBC | 18 (18.9) | 20 (21) |  |  | 5 (13.8) | 5 (13.8) |  |
| HER2-positive | 28 (29.4) | 35 (36.8) |  |  | 6 (16.6) | 14 (38.8) |  |
| **Neo-Bioscore score** |  |  |  |  |  |  |  |
| Median (range) | 4 (1-5) | 3 (1-6) |  |  | 3 (1-6) | 3 (0-7) |  |
| 1-3 | 51 (53.6) | 49 (51.5) | 0.771 |  | 17 (47.2) | 19 (52.7) | 0.814 |
| 4-6 | 44 (46.3) | 46 (48.4) |  |  | 19 (52.7) | 17 (47.2) |  |
| **Type of primary surgery** |  |  |  |  |  |  |  |
| Mastectomy | 84 (88.4) | 78 (82.1) | 0.306 |  | 33 (91.6) | 34 (94.4) | 1 |
| BCS | 11 (11.5) | 17 (17.8) |  |  | 3 (8.3) | 2 (5.5) |  |
| **NAT regimens** |  |  |  |  |  |  |  |
| Taxanes | 18 (18.9) | 11 (11.5) | 0.008 |  | 4 (11.1) | 4 (11.1) | 0.768 |
| Anthracycline | 17 (17.8) | 0 (0) |  |  | 11 (30.5) | 14 (38.8) |  |
| Taxanes + Anthracycline | 56 (58.9) | 83 (87.3) |  |  | 20 (55.5) | 16 (44.4) |  |
| Endocrine Therapy | 4 (4.2) | 1 (1) |  |  | 1 (2.7) | 2 (5.5) |  |
| **Response to NAT** |  |  |  |  |  |  |  |
| pCR | 16 (16.8) | 16 (16.8) | 0.874 |  | 0 (0) | 2 (5.5) | 0.41 |
| PR | 68 (71.5) | 70 (73.6) |  |  | 30 (83.3) | 29 (80.5) |  |
| SD | 7 (7.3) | 7 (7.3) |  |  | 4 (11.1) | 2 (5.5) |  |
| PD | 4 (4.2) | 2 (2.1) |  |  | 2 (5.5) | 3 (8.3) |  |
| **Adjuvant chemotherapy** |  |  |  |  |  |  |  |
| No | 38 (40) | 76 (80) | ＜0.001 |  | 11 (30.5) | 9 (25) | 0.793 |
| Yes | 57 (60) | 19 (20) |  |  | 25 (69.4) | 27 (75) |  |
| **Adjuvant chemotherapy regimens** |  |  |  |  |  |  |  |
| Taxanes | 27 (47.3) | 2 (10.5) | 0.002 |  | 12 (48) | 12 (44.4) | 0.414 |
| Anthracycline | 8 (14) | 0 (0) |  |  | 4 (16) | 1 (3.7) |  |
| Taxanes + Anthracycline | 12 (21) | 8 (42.1) |  |  | 8 (32) | 12 (44.4) |  |
| Other | 10 (17.5) | 9 (47.3) |  |  | 1 (4) | 2 (7.4) |  |
| **Targeted therapy in HER2+** |  |  |  |  |  |  |  |
| No | 6 (21.4) | 8 (22.8) | 0.892 |  | 3 (50) | 4 (28.5) | 0.357 |
| Yes | 22 (78.5) | 27 (77.1) |  |  | 3 (50) | 10 (71.4) |  |
| **RNI** |  |  |  |  |  |  |  |
| No | 6 (6.3) | 4 (4.2) | 0.747 |  | 0 (0) | 1 (2.7) | 1 |
| Yes | 89 (93.6) | 91 (95.7) |  |  | 36 (100) | 35 (97.2) |  |
| **IMN RT** |  |  |  |  |  |  |  |
| No | 55 (57.8) | 46 (48.4) | 0.245 |  | 15 (41.6) | 18 (50) | 0.637 |
| Yes | 40 (42.1) | 49 (51.5) |  |  | 21 (58.3) | 18 (50) |  |

Abbreviations: TTR=time to initiation of adjuvant radiotherapy; HR=hormone receptor; HER2= human epidermal growth factor receptor 2, IQR=interquartile range, BCS=breast conserving surgery; NAT=neoadjuvant treatment; pCR=pathological complete response; PR=partial response; SD=stable disease; PD=progressive disease; RNI=regional nodal irradiation; IMN=internal mammary nodes; RT=radiotherapy.**Table S2.** **Univariable analysis of potential risk factors for survival outcomes**

|  | **No. of patients** | **LRRFS** | | | |  | **DMFS** | | | |  | **RFS** | | | |  | **BCSS** | | | |
| --- | --- | --- | --- | --- | --- | --- | --- | --- | --- | --- | --- | --- | --- | --- | --- | --- | --- | --- | --- | --- |
|  |  | **No. of events** | **HR** | **95% CI** | **P value** |  | **No. of events** | **HR** | **95% CI** | **P value** |  | **No. of events** | **HR** | **95% CI** | **P value** |  | **No. of events** | **HR** | **95% CI** | **P value** |
| **TTR (weeks)** |  |  |  |  |  |  |  |  |  |  |  |  |  |  |  |  |  |  |  |  |
| 11-20 | 145 | 5 | 1 | - |  |  | 20 | 1 | - |  |  | 21 | 1 | - |  |  | 12 | 1 | - |  |
| ≤ 10 | 124 | 11 | 2.69 | 0.93-7.74 | 0.07 |  | 35 | 2.18 | 1.26-3.77 | 0.01 |  | 37 | 2.22 | 1.3-3.79 | <0.01 |  | 22 | 2.17 | 1.07-4.38 | 0.03 |
| ＞20 | 46 | 2 | 1.25 | 0.24-6.45 | 0.79 |  | 14 | 2.32 | 1.17-4.59 | 0.02 |  | 14 | 2.14 | 1.09-4.22 | 0.03 |  | 6 | 1.46 | 0.55-3.89 | 0.45 |
| **Age** (years) |  |  |  |  |  |  |  |  |  |  |  |  |  |  |  |  |  |  |  |  |
| as continuous | 315 | 18 | 1.03 | 1-1.07 | 0.08 |  | 69 | 1.01 | 0.99-1.03 | 0.39 |  | 71 | 1.01 | 0.99-1.03 | 0.34 |  | 40 | 1.01 | 0.99-1.04 | 0.29 |
| **Menopausal status** |  |  |  |  |  |  |  |  |  |  |  |  |  |  |  |  |  |  |  |  |
| Pre/peri-menopausal | 157 | 6 | 1 |  |  |  | 34 | 1 |  |  |  | 35 | 1 |  |  |  | 18 | 1 |  |  |
| Menopausal | 158 | 12 | 2.05 | 0.77-5.47 | 0.15 |  | 34 | 0.98 | 0.61-1.57 | 0.92 |  | 36 | 1.02 | 0.64-1.62 | 0.94 |  | 21 | 1.16 | 0.62-2.17 | 0.65 |
| **cT Stage** |  |  |  |  |  |  |  |  |  |  |  |  |  |  |  |  |  |  |  |  |
| T0-1 | 58 | 2 | 1 |  |  |  | 9 | 1 |  |  |  | 9 | 1 |  |  |  | 6 | 1 |  |  |
| T2 | 186 | 9 | 1.45 | 0.31-6.69 | 0.64 |  | 37 | 1.34 | 0.65-2.78 | 0.43 |  | 40 | 1.48 | 0.72-3.05 | 0.29 |  | 21 | 1.14 | 0.46-2.82 | 0.78 |
| T3 | 46 | 5 | 3.64 | 0.71-18.76 | 0.12 |  | 12 | 2.02 | 0.85-4.8 | 0.11 |  | 12 | 2.03 | 0.86-4.82 | 0.11 |  | 7 | 1.59 | 0.53-4.72 | 0.41 |
| T4 | 25 | 2 | 3.08 | 0.43-21.95 | 0.26 |  | 11 | 4.06 | 1.68-9.82 | <0.01 |  | 11 | 4.13 | 1.71-9.98 | <0.01 |  | 6 | 3.55 | 1.14-11.05 | 0.03 |
| **cN Stage** |  |  |  |  |  |  |  |  |  |  |  |  |  |  |  |  |  |  |  |  |
| N0 | 39 | 1 | 1 |  |  |  | 5 | 1 |  |  |  | 7 | 1 |  |  |  | 4 | 1 |  |  |
| N1 | 152 | 8 | 2.11 | 0.26-16.85 | 0.48 |  | 28 | 1.54 | 0.59-3.99 | 0.38 |  | 28 | 1.09 | 0.48-2.49 | 0.84 |  | 17 | 1.16 | 0.39-3.43 | 0.80 |
| N2 | 93 | 4 | 1.9 | 0.21-17.04 | 0.57 |  | 19 | 1.99 | 0.74-5.35 | 0.17 |  | 20 | 1.49 | 0.63-3.53 | 0.37 |  | 11 | 1.78 | 0.56-5.64 | 0.33 |
| N3 | 31 | 5 | 8.38 | 0.97-72.07 | 0.05 |  | 17 | 7.54 | 2.77-20.56 | <0.01 |  | 17 | 5.49 | 2.26-13.31 | <0.01 |  | 8 | 5.03 | 1.5-16.91 | 0.01 |
| **ypT Stage** |  |  |  |  |  |  |  |  |  |  |  |  |  |  |  |  |  |  |  |  |
| T0-is | 63 | 0 | - |  | 0.97 |  | 4 | 1 |  |  |  | 5 | 1 |  |  |  | 4 | 1 |  |  |
| T1 | 128 | 9 | 1 |  |  |  | 27 | 3.43 | 1.2-9.82 | 0.02 |  | 29 | 3.01 | 1.16-7.78 | 0.02 |  | 14 | 1.57 | 0.51-4.76 | 0.43 |
| T2 | 97 | 4 | 0.59 | 0.18-1.91 | 0.38 |  | 24 | 4.27 | 1.48-12.31 | 0.01 |  | 24 | 3.38 | 1.29-8.87 | 0.01 |  | 12 | 1.88 | 0.61-5.83 | 0.28 |
| T3 | 21 | 5 | 5.33 | 1.77-16.05 | <0.01 |  | 11 | 15.58 | 4.95-49.02 | <0.01 |  | 11 | 12.47 | 4.32-35.97 | <0.01 |  | 7 | 8.06 | 2.35-27.62 | <0.01 |
| T4 | 6 | 0 | - |  | 0.99 |  | 3 | 18.39 | 4.11-82.33 | <0.01 |  | 3 | 15.03 | 3.59-63.04 | <0.01 |  | 3 | 10.58 | 2.36-47.39 | <0.01 |
| **ypN Stage** |  |  |  |  |  |  |  |  |  |  |  |  |  |  |  |  |  |  |  |  |
| N0 | 109 | 2 | 1 |  |  |  | 8 | 1 |  |  |  | 10 | 1 |  |  |  | 6 | 1 |  |  |
| N1 | 85 | 2 | 1.3 | 0.18-9.26 | 0.79 |  | 13 | 2.16 | 0.89-5.21 | 0.09 |  | 13 | 1.70 | 0.75-3.88 | 0.21 |  | 6 | 0.42 | 0.22-0.81 | 0.01 |
| N2 | 69 | 5 | 4.43 | 0.86-22.85 | 0.08 |  | 18 | 4.38 | 1.9-10.07 | <0.01 |  | 19 | 3.72 | 1.73-7.99 | <0.01 |  | 13 | 0.52 | 0.27-1.01 | 0.05 |
| N3 | 52 | 9 | 11.64 | 2.51-53.91 | <0.01 |  | 30 | 12.17 | 5.57-26.57 | <0.01 |  | 30 | 9.97 | 4.86-20.42 | <0.01 |  | 15 | 1.67 | 1-2.78 | 0.05 |
| **Histological grade** |  |  |  |  |  |  |  |  |  |  |  |  |  |  |  |  |  |  |  |  |
| I | 9 | 1 | 1 |  |  |  | 1 | 1 |  |  |  | 1 | 1 |  |  |  | 1 | 1 |  |  |
| II | 149 | 4 | 0.23 | 0.03-2.04 | 0.19 |  | 28 | 1.66 | 0.23-12.23 | 0.62 |  | 30 | 1.79 | 0.24-13.09 | 0.57 |  | 16 | 0.99 | 0.13-7.47 | 0.99 |
| III | 157 | 13 | 0.75 | 0.1-5.7 | 0.78 |  | 40 | 2.48 | 0.34-18.04 | 0.37 |  | 41 | 2.57 | 0.35-18.69 | 0.35 |  | 23 | 1.50 | 0.2-11.13 | 0.69 |
| **HR status** |  |  |  |  |  |  |  |  |  |  |  |  |  |  |  |  |  |  |  |  |
| Negative | 133 | 12 | 1 |  |  |  | 29 | 1 |  |  |  | 31 | 1 |  |  |  | 26 | 1 |  |  |
| Positive | 182 | 6 | 0.31 | 0.12-0.84 | 0.02 |  | 40 | 0.87 | 0.54-1.41 | 0.58 |  | 41 | 0.82 | 0.51-1.3 | 0.40 |  | 14 | 0.30 | 0.16-0.58 | <0.01 |
| **HER2 status** |  |  |  |  |  |  |  |  |  |  |  |  |  |  |  |  |  |  |  |  |
| Negative | 200 | 14 | 1 |  |  |  | 52 | 1 |  |  |  | 55 | 1 |  |  |  | 28 | 1 |  |  |
| Positive | 115 | 4 | 0.47 | 0.15-1.43 | 0.18 |  | 17 | 0.52 | 0.3-0.9 | 0.02 |  | 17 | 0.49 | 0.29-0.85 | 0.01 |  | 12 | 0.74 | 0.38-1.45 | 0.38 |
| **Ki67 (%)** |  |  |  |  |  |  |  |  |  |  |  |  |  |  |  |  |  |  |  |  |
| as continuous |  |  | 1.02 | 1-1.04 | 0.05 |  |  | 1 | 0.99-1.01 | 0.54 |  |  | 1.01 | 1-1.02 | 0.32 |  |  | 1.01 | 1-1.03 | 0.11 |
| ≤ 14 | 63 | 4 | 1 |  |  |  | 14 | 1 |  |  |  | 14 | 1 |  |  |  | 4 | 1 |  |  |
| >14 | 221 | 11 | 0.79 | 0.25-2.5 | 0.69 |  | 45 | 0.92 | 0.5-1.67 | 0.78 |  | 47 | 0.97 | 0.53-1.76 | 0.91 |  | 27 | 2.14 | 0.75-6.12 | 0.16 |
| **Molecular subtype** |  |  |  |  |  |  |  |  |  |  |  |  |  |  |  |  |  |  |  |  |
| Luminal | 131 | 5 | 1 |  |  |  | 33 | 1 |  |  |  | 33 | 1 |  |  |  | 12 | 1 |  |  |
| TNBC | 69 | 9 | 4.094 | 1.369-12.242 | 0.012 |  | 21 | 1.521 | 0.873-2.65 | 0.138 |  | 22 | 1.65 | 0.965-2.822 | 0.067 |  | 17 | 3.899 | 1.818-8.36 | 0.01 |
| HER2-positive | 115 | 4 | 0.912 | 0.245-3.398 | 0.891 |  | 19 | 0.604 | 0.334-1.091 | 0.095 |  | 19 | 0.59 | 0.328-1.063 | 0.079 |  | 12 | 1.352 | 0.596-3.071 | 0.47 |
| **Type of primary surgery** |  |  |  |  |  |  |  |  |  |  |  |  |  |  |  |  |  |  |  |  |
| BCS | 44 | 1 | 1 |  |  |  | 2 | 1 |  |  |  | 3 | 1 |  |  |  | 0 | 1 |  |  |
| Mastectomy | 271 | 17 | 3.07 | 0.41-23.1 | 0.28 |  | 67 | 6.72 | 1.65-27.42 | 0.01 |  | 69 | 4.62 | 1.45-14.67 | 0.01 |  | 40 | 26.62 | 0.71-999.78 | 0.08 |
| **Response to NAT** |  |  |  |  |  |  |  |  |  |  |  |  |  |  |  |  |  |  |  |  |
| PD | 13 | 4 | 1 |  |  |  | 7 | 1 |  |  |  | 7 | 1 |  |  |  | 2 | 1 |  |  |
| pCR | 48 | 0 | / | / | 0.97 |  | 0 | / | / | 0.93 |  | 1 | 0.02 | 0.003-0.19 | <0.01 |  | 0 | / | / | 0.96 |
| PR | 233 | 13 | 0.13 | 0.04-0.4 | <0.01 |  | 58 | 0.31 | 0.14-0.68 | <0.01 |  | 60 | 0.33 | 0.15-0.72 | 0.01 |  | 37 | 0.73 | 0.18-3.03 | 0.66 |
| SD | 21 | 1 | 0.11 | 0.01-0.98 | 0.048 |  | 4 | 0.23 | 0.07-0.77 | 0.02 |  | 4 | 0.23 | 0.07-0.79 | 0.02 |  | 1 | 0.22 | 0.02-2.44 | 0.22 |
| **Targeted therapy** |  |  |  |  |  |  |  |  |  |  |  |  |  |  |  |  |  |  |  |  |
| No | 226 | 16 | 1 |  |  |  | 59 | 1 |  |  |  | 62 | 1 |  |  |  | 32 | 1 |  |  |
| Yes | 89 | 2 | 0.31 | 0.07-1.36 | 0.12 |  | 10 | 0.41 | 0.21-0.81 | 0.01 |  | 10 | 0.40 | 0.2-0.78 | 0.01 |  | 8 | 0.72 | 0.33-1.56 | 0.40 |
| **RNI** |  |  |  |  |  |  |  |  |  |  |  |  |  |  |  |  |  |  |  |  |
| No | 18 | 1 | 1 |  |  |  | 1 | 1 |  |  |  | 3 | 1 |  |  |  | 1 | 1 |  |  |
| Yes | 297 | 17 | 1.15 | 0.15-8.66 | 0.89 |  | 68 | 5.07 | 0.7-36.54 | 0.11 |  | 69 | 1.67 | 0.53-5.31 | 0.38 |  | 39 | 2.67 | 0.37-19.42 | 0.33 |
| **IMN RT** |  |  |  |  |  |  |  |  |  |  |  |  |  |  |  |  |  |  |  |  |
| No | 160 | 9 | 1 |  |  |  | 27 | 1 |  |  |  | 29 | 1 |  |  |  | 12 | 1 |  |  |
| Yes | 155 | 9 | 1.06 | 0.42-2.66 | 0.91 |  | 42 | 1.72 | 1.06-2.79 | 0.03 |  | 43 | 1.63 | 1.02-2.61 | 0.04 |  | 28 | 2.55 | 1.3-5.02 | <0.01 |

Abbreviations: LRRFS=locoregional recurrence free survival; DMFS=distant metastasis free survival; RFS=recurrence free survival; BCSS=breast cancer specific survival; HR=hazard ratio; CI=confidence interval

**Table S3.** **Adjusted hazard ratio of survival outcomes by TTR in the multivariable cox proportional hazards model among matched cohorts**

|  | **LRRFS** | | |  | **DMFS** | | |  | **RFS** | | |  | **BCSS** | | |
| --- | --- | --- | --- | --- | --- | --- | --- | --- | --- | --- | --- | --- | --- | --- | --- |
| **Parameters** | **HR** | **95% CI** | **P value** |  | **HR** | **95% CI** | **P value** |  | **HR** | **95% CI** | **P value** |  | **HR** | **95% CI** | **P value** |
| **Matched cohort** **1 (N=190)** | | | | | | | | | | | | | | | |
| **TTR (weeks)** |  |  |  |  |  |  |  |  |  |  |  |  |  |  |  |
| 11-20 | 1 | / | 0.06 |  | 1 | / | 0.006 |  | 1 | / | 0.005 |  |  |  | 0.698 |
| ≤ 10 | 4.38 | 0.93-20.52 |  |  | 3.09 | 1.38-6.9 |  |  | 2.99 | 1.38-6.43 |  |  | 1.19 | 0.47-2.99 |  |
| **Response to NAT** |  |  |  |  |  |  |  |  |  |  |  |  |  |  |  |
| pCR |  |  |  |  |  |  |  |  | 1 | / |  |  |  |  |  |
| PR |  |  |  |  |  |  |  |  | 7.86 | 1.02-60.13 | 0.047 |  |  |  |  |
| SD |  |  |  |  |  |  |  |  | 3.24 | 0.31-33.68 | 0.325 |  |  |  |  |
| PD |  |  |  |  |  |  |  |  | 4.86 | 0.28-84.03 | 0.277 |  |  |  |  |
| **Adjuvant chemotherapy** |  |  |  |  |  |  |  |  |  |  |  |  |  |  |  |
| No | 1 | / | 0.026 |  | 1 | / | 0.025 |  |  |  |  |  |  |  |  |
| Yes | 8.11 | 1.28-51.11 |  |  | 2.55 | 1.12-5.82 |  |  |  |  |  |  |  |  |  |
| **IMN RT** |  |  |  |  |  |  |  |  |  |  |  |  |  |  |  |
| No |  |  |  |  | 1 | / | 0.029 |  | 1 | / | 0.034 |  | 1 | / | 0.006 |
| Yes |  |  |  |  | 2.22 | 1.08-4.58 |  |  | 2.08 | 1.05-4.12 |  |  | 3.89 | 1.48-10.2 |  |
| **Matched cohort 2 (N=72)** | | | | | | | | | | | | | | | |
| **TTR (weeks)** |  |  |  |  |  |  |  |  |  |  |  |  |  |  |  |
| 11-20 | 1 | / | 0.615 |  | 1 | / | 0.276 |  | 1 | / | 0.276 |  | 1 | / | 0.67 |
| ＞20 | 0.4 | 0.01-13.44 |  |  | 1.89 | 0.6-5.98 |  |  | 1.89 | 0.6-5.98 |  |  | 0.7 | 0.14-3.47 |  |
| **cN Stage** |  |  |  |  |  |  |  |  |  |  |  |  |  |  |  |
| N0 |  |  |  |  |  |  |  |  |  |  |  |  |  |  |  |
| N1 |  |  |  |  |  |  |  |  |  |  |  |  | 0.33 | 0.05-2.23 | 0.26 |
| N2 |  |  |  |  |  |  |  |  |  |  |  |  | 1.15 | 0.13-9.96 | 0.896 |
| N3 |  |  |  |  |  |  |  |  |  |  |  |  | 40.86 | 1.06-1573.22 | 0.046 |

Note: Variables in the model include cT, cN , response to NAC, type of primary surgery, administration of adjuvant chemotherapy, targeted therapy, and delivery of IMN RT.

Only TTR and variables identified as independent prognostic factors in the multivariable analysis were showed in this table.
